# Severe Gastrointestinal Bleeding in a Patient With Subvalvular Aortic Stenosis Treated With Thalidomide and Octreotide: Bridging to Transcoronary Ablation of Septal Hypertrophy

**DOI:** 10.14740/jocmr2321w

**Published:** 2015-09-25

**Authors:** Helene S. Hvid-Jensen, Steen H. Poulsen, Jorgen S. Agnholt

**Affiliations:** a Department of Hepato-Gastroenterology V and Department of Cardiology, Aarhus University Hospital, DK-8000 Aarhus, Denmark

**Keywords:** Subvalvular aortic obstruction, Hypertrophic cardiomyopathy, Angiodysplasia, Gastrointestinal bleeding, Thalidomide, Sandostatin, Heyde’s syndrome

## Abstract

Gastrointestinal bleeding (GB) due to angiodysplasias can cause severe, recurrent bleeding, especially in elderly patients. Angiodysplastic bleedings in the gastrointestinal tract have been associated with aortic stenosis and, more recently, hypertrophic obstructive cardiomyopathy, caused by an acquired coagulopathy known as Heyde’s syndrome. Multiple factors are involved in the pathogenesis of angiodysplastic bleeding including genetic factors and increased levels of vascular intestinal growth factor at tissue levels. Endoscopic coagulation therapy is the primary treatment but often fails to resolve bleeding, especially in patients with large numbers of angiodysplasias. In patients with aortic stenosis and GB, the main treatment is aortic valve replacement but the patients may be unfit to undergo surgery due to the complicating anemia. In this case story, we present a patient with severe, GB due to hypertrophic subvalvular obstructive cardiomyopathy. Endoscopic procedures with argon beaming were performed without effect on bleeding. The patient was treated with a combination of both thalidomide and octreotide. Within 3 months, the patient recovered from the anemia and was able to undergo transcoronary ethanol ablation. No further bleeding episodes occurred, and thalidomide and octreotide were arrested. To our knowledge, this case report is the first to describe how this new drug combination therapy is an effective treatment of GB from angiodysplasias and can be used to bridge to surgical or endovascular treatment.

## Introduction

Angiodysplasias are a well-known cause of gastrointestinal bleeding (GB) accounting for up to 6% of patients hospitalized with GB [1, 2]. The prevalence is especially high among elderly patients [1, 3], and often the patients have been through extensive examinations and numerous hospitalizations before diagnosis is made. Furthermore, up to 40% of the bleeding episodes originate from small intestine angiodysplasias [4], previously making it difficult to diagnose and access for treatment. However, capsule endoscopy and double balloon or spiral endoscopy have improved diagnosis significantly, although treatment may still be a task since re-bleeding after argon beaming of angiodysplastic lesions is frequently observed [5].

Aortic valve stenosis (AS) and, more recently, hypertrophic obstructive cardiomyopathy (HOCM) are associated with anemia due to GB from angiodysplasias [1, 6-8]. This uncommon condition, known as Heyde’s syndrome, was first described by Edward Heyde in 1958 in a case series of 10 patients with AS and massive intestinal bleeding. Since the initial discussion of association or coincidence between AS and GB, four main theories regarding the pathophysiological mechanism have emerged: acquired von Willebrand factor (vWF) deficiency, mucosal ischemia, age-related degeneration or perhaps genetic factors.

With the acquired form of von Willebrand disease, the defect is causing bleeding in places with high velocity blood flow. vWF is a protein multimer of different sizes. The largest multimers are responsible for platelet adhesion and hemostasis when bleeding occurs in places with high velocity blood flow, as found in small intestinal arterioles. Vascular abnormalities, such as aortic valve obstruction or left ventricular outflow obstruction, with high shear stress cause degradation of the vWF molecule and impaired hemostasis. Several studies have shown how aortic valve replacement [4, 8, 9] and, more recently, surgical septal myectomy [2], result in normalization of the vWF multimer pattern and resolution of anemia/bleeding. However, a case-control study by Hoog et al [1] has demonstrated similar changes in patient’s bleeding from other causes than angiodysplasia suggesting that bleeding from angioectasia is caused by multiple factors.

AS and GB are most frequent in elderly patients (> 60 years) supporting the hypothesis of regenerative changes and ischemia. The narrowing of the aortic valve reduces the pulsatile flow, which might reduce mucosal perfusion, inducing mucosal ischemia. As a consequence, increased levels of vascular intestinal growth factor (VEGF) have been observed at tissue levels, facilitating the development of new vessels/angioectasias. This observation may have paved the way for the treatment with thalidomide (see below). Genetic factors may also participate in the pathophysiological mechanisms of AS and GB, probably due to a defect in supporting collagen IV.

In patients with AS and GB, the main treatment is aortic valve replacement. In cases of severe GB, it may be necessary to “bridge” the patient with medical and/or endoscopic treatments in order to stabilize the patient before valve replacement.

In the actual case report, we present a patient with severe GB from angiodysplasias associated with HOCM where medical “bridging” therapy was necessary to make the patient suitable for either transcoronary alcohol ablation or surgical subvalvular myomectomy.

## Case Report

A 67-year-old woman was acutely admitted to the cardiological department presenting with angina, dyspnea (NYHA II), melena, hypotension and anemia (hemoglobin (Hgb) 3.2 mmol/L (reference: 7.3 - 9.5)). She had a history of paroxystic atrial fibrillation treated with amiodarone and an HOCM due to a displaced artificial mitral valve causing a subvalvular aortic stenosis diagnosed 6 months prior to the admission. An echocardiography had revealed a high left ventricular outflow tract (LVOT) obstruction with a gradient of 140 mm Hg (normal values < 30 mm Hg).

**Figure 1:**
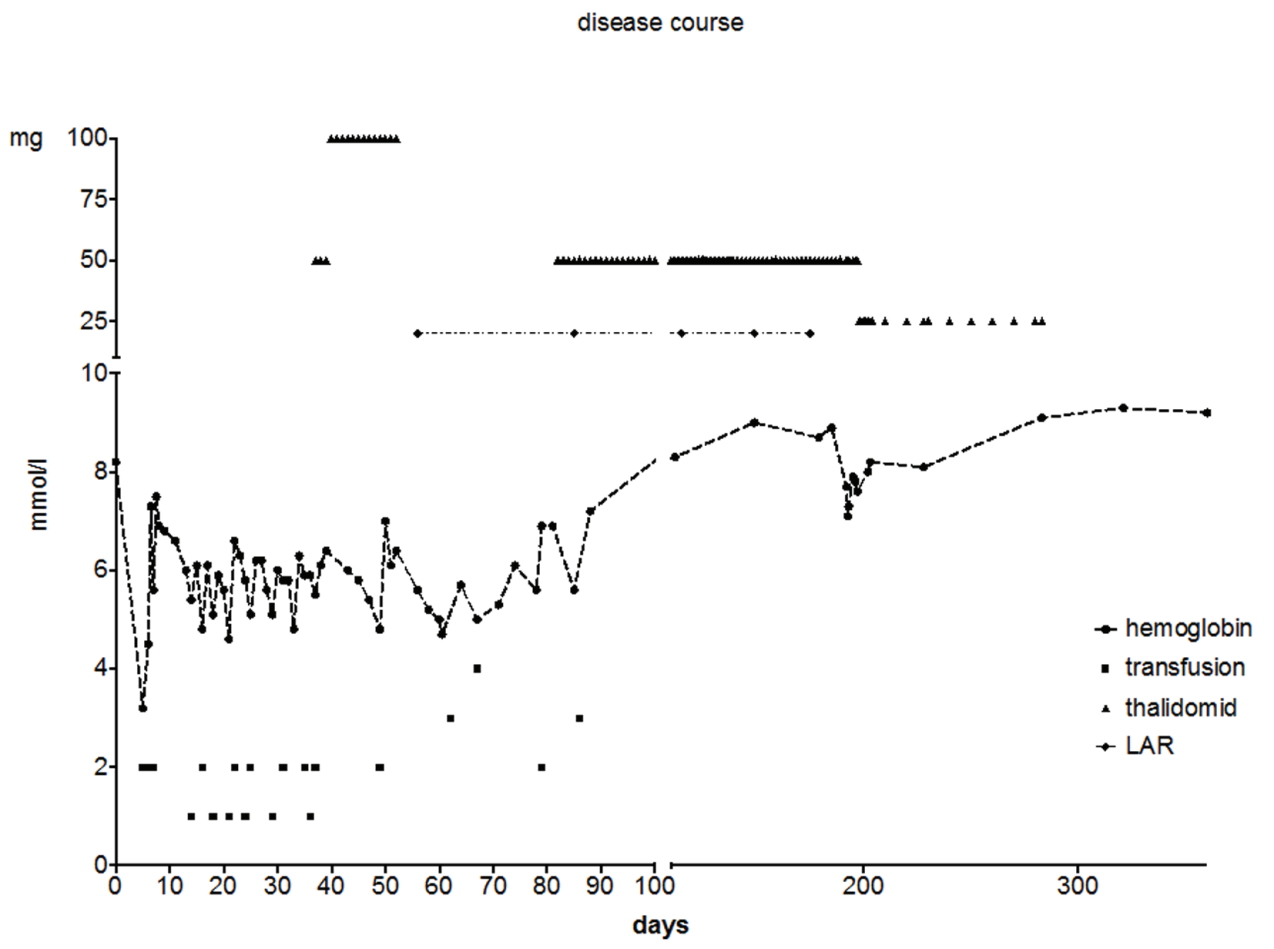
Disease course of the patient. The figure shows the relationship between treatment with thalidomide and octreotide, number of transfusions and hemoglobin. Spiral endoscopy was performed at day 25. Angiodysplasias were treated with argon beaming. Hemoglobin: mmol/L; LAR: Sandostatin^®^ LAR (octreotide). At day 500, the patient was discharged from the outpatient clinic of the Department of Hepato-Gastroenterology with a hemoglobin of 9.3 mmol/L.

An acute gastroscopy revealed gastritis and hematin spots. Besides a few spots with slight bleeding were observed and warfarin therapy was discontinued (instead she was treated with low molecular heparin due to the artificial mitral valve). The patient continued having episodes of melena and the anemia persisted despite numerous blood transfusions. From the admission and during the next 34 days, she received 24 transfusions. Renewed gastroscopy, colonoscopy and CT angiography did not reveal any source of bleeding. A capsule endoscopy showed multiple angiodysplasias in the small intestine. During an oral spiral endoscopy, the bleeding elements were visualized from the Treitz ligament and in the proximal part of the jejunum. In the distal jejunum and proximal ileum, we did not disclose any angiodysplasias. They were treated with argon beaming. However, bleeding persisted and the patient received continuous blood transfusions. Two weeks treatment with thalidomide 50/100 mg per day was initiated with no significant effect on Hgb levels. Thalidomide was arrested for 4 weeks due to induction of bradycardia and QT prolongation. We considered it was a side effect of the thalidomide treatment. At an interdisciplinary conference, the possibility of Heyde’s syndrome was raised and cardiac surgery was considered, despite the patient’s severe physical condition, in order to cease GB. As a last resort, after an octreotide test-dosis of three times 50 μg (which were well tolerated), the patient was treated with Sandostatin^®^ (octreotide) slow release formulation 20 mg injections every 4 weeks (Fig. 1). The demand for transfusion was reduced after the combined treatment with argon beaming and medical treatment. Still, a need for transfusion persisted, and further 11 transfusions were given during the next 42 days (Fig. 1). A renewed capsule endoscopy showed three uncharacteristic areas of bleeding but no signs of angiodysplasias. Still repeating blood transfusions were necessary in order to maintain stable Hgb levels. For this reason, treatment with thalidomide was resumed at a lower dose (50 mg per day) alongside with octreotide treatment (Fig. 1). After 2 weeks at combined treatment, the Hgb was normalized and no further transfusions were needed during an observation period of 1 year. After 3 months with normal Hgb, the subvalvular hypertrophy was treated with TACE (alcohol septal ablation) with a positive effect on the patient’s cardiac symptoms. Treatment with octreotide was continued until 1 month after the TACE treatment where the gradient was reduced from more than 100 mm Hg to 50 mm Hg. Thalidomide was slowly tapered (50 mg every second day → 50 mg twice a week). Five months after surgery, an echocardiography revealed a reduction of the LVOT gradient to a near normal level (40 mm Hg) and thalidomide was therefore halted. Twelve months following treatment, the patient has had no symptoms of GB and Hgb has stabilized (8.8 - 9 mm Hg) with no need for further blood transfusions.

## Discussion

In this case report, severe, recurrent GB from angiodysplasias in a patient with left ventricular subvalvular obstruction was efficiently treated with thalidomide and octreotide as bridging to cardiac surgery. During admission, she received no less than 35 blood transfusions as a result of recurrent GB from angiodysplastic lesions.

The association between aortic stenosis and, more recently, HOCM/subvalvular aortic stenosis [6, 10] and GB from angiodysplasia is known as Heyde’s syndrome. The pathophysiology of Heyde’s syndrome as an acquired deficiency of vWF is well described but difficult to treat. Furthermore, several studies [1, 6, 9] conclude that multiple pathophysiological mechanisms are involved in the pathogenesis of angiodysplastic bleeding, including an increased production of VEGF, due to a reduced pulsatile flow impairing intestinal blood flow. At present, conventional therapies including local endoscopic ablation, argon beaming and surgical resection are often ineffective in preventing recurrent GB, and may be associated with severe complications. Several studies [2, 4, 10] support that cardiac surgery can prevent recurrent bleeding. Aortic valve replacement in patients with aortic stenosis usually resolves GB. Similar results are reported in patients with HOCM after surgical septal myectomy [2, 10]. However, the patients, as in this case, are often in too poor a condition to undergo surgery.

In this case, the patient was treated with a combination of thalidomide and octreotide, causing resolution of GB. This combination of therapy is not previously described in literature. Octreotide is known to reduce blood loss from intestinal angiodysplasias through vasoconstriction. Thalidomide inhibits the expression of VEGF, causing an anti-angiogenetic effect. The fact that treatment with thalidomide had a positive effect on GB supports the thesis that production of VEGF plays a significant role in the pathogenesis of angiodysplastic bleeding.

Furthermore, this case demonstrates how thalidomide, in combination with octreotide, is an effective and relatively safe method of therapy against recurrent GB. Endoscopic intervention is always relevant, but medical treatment should be considered in those cases where bleeding is severe and the patient is unfit to undergo surgical intervention. In these situations, we suggest combinational therapy with thalidomide and octreotide as “bridging” to surgical treatment.
